# Impacts of a Prescribed Physical Activity Program for People with Chronic Diseases Living in Community Settings in France

**DOI:** 10.3390/ijerph21080966

**Published:** 2024-07-24

**Authors:** Christophe Martinez, Aurélie Goncalves, Olivier Coste, Sarah Pabion, Elodie Charbonnier

**Affiliations:** 1 UNIV. NIMES, APSY-V, F-30021 Nîmes Cedex 1, France; christophe.martinez1@unimes.fr (C.M.);; 2 Nîmes Sport Santé, 30000 Nîmes, France; maisonsportsantenimes@gmail.com; 3 Délégations Régionales Académiques à la Jeunesse à L’engagement et aux Sports, 34000 Montpellier, France

**Keywords:** prescription exercise, chronic disease, obesity, adapted physical activity

## Abstract

Background: Sedentary behavior and physical inactivity are modifiable risk factors at the forefront of prevention and health promotion strategies. The health benefits of physical activity (PA) have been widely demonstrated in chronic diseases and have led to the prescription of adapted PA. To date, French scientific data are insufficient to evaluate the effectiveness of prescribing adapted PA. This study aimed (1) to evaluate the effectiveness of a community-based program and (2) to identify patient characteristics at inclusion that could be associated with improved post-program anthropometric data. Methods: Our sample was composed of 113 participants with a chronic disease (83.18% women) with a mean age of 55.4 ± 13.9 years. Participants benefited from an 8-week adapted PA program. All participants were evaluated at the beginning and end of the program by anthropometric measurements, a subjective measure of the level of PA and a measure of physical condition. Results: Almost 86% of the participants were overweight and two-thirds were obese. Statistical analyses showed a significant improvement in physical condition, expressed by a better cardiorespiratory endurance (up to +14% for a 2 min walk test; M_T0_ = 78.1 m vs. M_T1_ = 89 m; *p* < 0.001 with a 2 min walk test), improved flexibility (+12.5%; M_T0_ = 2.4 vs. M_T1_ = 2.7; *p* < 0.001), and increased muscle strength in the lower limbs (+22.7%; M_T0_ = 11.9 vs. M_T1_ = 14.6; *p* < 0.001). The level of physical activity increased significantly for all participants (57.52% of inactive individuals at T0 vs. 5.31% at T1; *p* = 0.004). Correlational analyses revealed that the decrease in BMI and weight throughout the program correlated positively with age (r = 0.252 and *p* = 0.007, and r = 0.247 and *p* = 0.008, respectively) and negatively with BMI from baseline (r = −0.271; *p* = 0.004). Conclusions: The key points of this community-based PA program are the following: (1) It improves participants’ physical condition. (2) It improves anthropometric parameters. (3) It modifies physical activity behavior. Furthermore, in the context of the program set up specifically for this purpose, it would appear that elderly and overweight people are more likely to exhibit beneficial effects on anthropometric parameters than younger participants or those with a high level of obesity. However, these results need to be confirmed by a long-term evaluation of the effectiveness of such devices.

## 1. Introduction

According to the World Health Organization (WHO), non-communicable diseases (NCDs) are long-term conditions that typically progress slowly and that significantly impact the daily lives of patients and their families. In 2023, all NCDs accounted for 74% of deaths worldwide, or 41 million deaths [1]. Many NCDs are caused by a short list of risk factors such as harmful use of alcohol, dietary sodium intake, tobacco use, raised blood pressure, diabetes, obesity, physical inactivity, and sedentary behavior. The physical inactivity term corresponds to an insufficient level of physical activity (PA) to meet present PA recommendations. It is important to distinguish it from sedentary behavior, which is any waking behavior characterized by an energy expenditure of 1.5 or lower metabolic equivalent (MET), while in a sitting, reclining, or lying posture [2].

The latest French report on physical inactivity and sedentary behavior in the general population is alarming [3]. Only 5% of adults are sufficiently physically active for it to be protective. Thus, 35.5 million adults are now exposed to high (but avoidable) health risks. Slightly more than one-third of adults spend more than 8 h per day participating in sedentary behavior, with a higher proportion among younger adults (42% of 18–44-year-olds) than older adults (31% of 45–64-year-olds). Regarding the PA WHO recommendations of 150 min (minimum) per week of moderate to vigorous intensity, 68% of adults do not meet the threshold, with a higher proportion among women (82% versus 57% among men). PA contributes to the prevention, management, and treatment of NCDs including cardiovascular diseases, diabetes, stroke, colon and breast cancers, osteoarthritis, osteoporosis, obesity, and mental and psychological illnesses [4]. This situation has led to the development of numerous referral programs to promote a more active lifestyle and less sedentary behavior for people with chronic diseases, both in European countries and in Canada, New Zealand, and the USA. The first such program was introduced in the UK during the 1990s as exercise referral schemes (ERSs). Depending on the country of origin, these programs may also be named physical activity referral schemes (PARSs) or chronic disease management (CDM). Despite the deployment of numerous PA programs, there is a continued lack of evidence for successful interventions in real-world settings, such as the community, as only a small number of interventions move from research into practice [5]. Consequently, translating efficacious interventions into practice within community settings is a major public health challenge.

In France, this lag between research and practice is all the more marked, as the prevention scheme called physical activity on prescription (or PAP) was only created in 2016, whereas the UK had established it 26 years earlier. Thanks to the law of modernization of the French health system (LOI n°2016-41 du 26 janvier 2016), physicians are authorized to formally prescribe participation in health-oriented physical activity programs to their patients. Prescribed PA programs correspond to PA and sports programs, adapted to the person’s abilities, and generally provided to vulnerable populations (e.g., elderly people, people with chronic diseases), for preventive, re-education, rehabilitation, reintegration, educational, and/or social participation objectives [6]. Several international reviews report an increase in PA levels and an improvement in various physical fitness parameters following PA prescription schemes [7,8,9].

Along the same lines, the prescription of PA has shown its value in the treatment of metabolic diseases, cardiac and pulmonary pathologies, muscular diseases, and cancer [10,11,12]. Despite these encouraging international data, to date, there are few French studies evaluating the effects of support systems following a PA prescription for individuals with a chronic disease. The French studies report an improvement in the distance covered in the six-minute walk test, in PA levels, and a significant weight loss of 2 kg after 6 months of accompaniment [13,14]. Unsurprisingly, weight is one of the indicators most often used alongside PA level to assess the effect of PA prescription schemes. Overweight and obesity are associated with an increased risk of morbidity and mortality [15]. Weight loss is a key indicator of health improvement [16], especially for people with chronic diseases [16,17]. Lifestyle interventions incorporating PA significantly improve weight and decrease cardio-metabolic risk factors in grade 2 and 3 obese subjects [18]. In addition, weight loss is positively associated with increased PA levels [19,20]. It therefore seems worthwhile to achieve weight loss or a reduction in Body Mass Index (BMI), thus encouraging the maintenance of an active lifestyle. Moreover, while there is a consensus in the literature on the beneficial effects of PA and PA prescription on health, no French study has focused on the parameters at program inclusion that could lead to forecasts of which participants would benefit most from the program in terms of anthropometric data.

This study encompassed two aims: first, to measure the effectiveness of a regional community scheme aimed at supporting people who have received a prescription for PA, to supplement the limited data available on adults from France on the subject; second, to identify patient characteristics at inclusion that could be associated with greater effectiveness of the program.

To fulfill the first objective, we hypothesize that completion of a PA program will result in the following:

**H1.**  *A significant improvement in participants’ physical condition (e.g., cardiorespiratory endurance, strength, flexibility).*

**H2.**  *An improvement in anthropometric measurements with, in particular, a loss of weight, BMI, and waist circumference.*

**H3.**  *A change in health-promoting behaviors through an increase in the level of PA and a reduction in sedentary behaviors.*

To reach the second objective, given that very little research has been carried out on the characteristics of the people for whom these programs are most successful, we make the following exploratory hypothesis (H4):

**H4.**  *Certain characteristics of the individuals on inclusion may be associated with greater program effectiveness.*

To determine this, three anthropometric indicators were considered: BMI, weight, and waist circumference.

## 2. Materials and Methods

### 2.1. Research Design

The “Bougez sur ordonnance” (BSO^®^—“Move on prescription”) scheme was created by the Occitanie region, as part of the introduction of PAP in France in 2016. This scheme is then implemented at local level by physical activity and sports associations. The “BSO” scheme is designed to promote PA among the sedentary, physically inactive, and chronically diseased population. In the context of this study, it was deployed in the community by the “Nîmes Sport Santé” association and provided in the towns of Nîmes and Vauvert, which are urban areas.

The absence of data in France on PAP schemes led us to analyze, with a retrospective study design, the data collected by the local association on all participants who wished to engage in the BSO scheme, at the outset of the PAP, during three seasons (2016/2017, 2017/2018, 2018/2019). Only the first 3 years could be collected before the onset of COVID-19, during which the practice of sport was prohibited by the public authorities for a long period of time in France.

### 2.2. Population

The sample size estimation was assessed using the G*Power 3.1.9.7 software (with a 1-beta power of 80% and an alpha risk of 5%). The number of participants, considering a medium effect size (Cohen’s d = 0.50) in the absence of previous data in the literature, was n = 28. To meet statistical requirements while anticipating the loss of participants, a larger number of participants was initially recruited.

**Figure 1:**
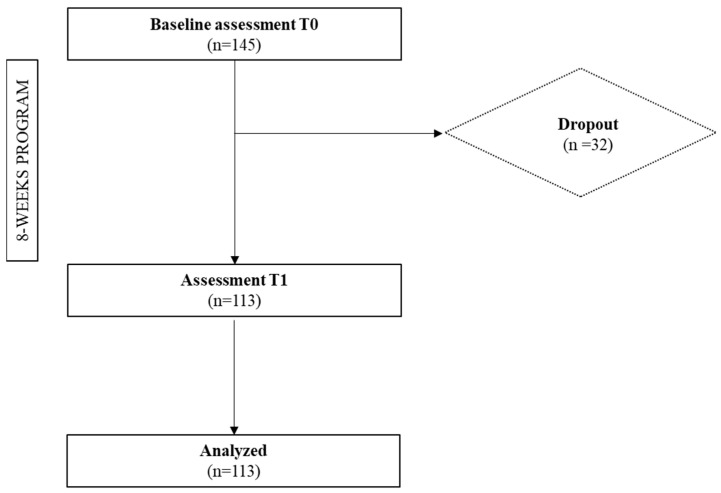
Flow diagram for study participants.

The association initially included 145 people (Figure 1). The study’s inclusion criteria were as follows: (1) to receive a PAP, (2) to agree to take part in the entire intervention, and (3) to agree to take part in the research protocol, which was evidenced by an information sheet and a consent form. The exclusion criteria were as follows: participants who came to the association without benefiting from a PAP, who did not wish to commit to the program as a whole, or who did not wish to take part in the research protocol. It is important to note that all the people met our inclusion criteria, so no one was excluded.

More than 22% (*n* = 32) of the participants stopped during the program for various reasons (e.g., schedule change no longer allowing attendance at activities, worsening of the disease, etc.). Thus, our final sample consisted of 113 participants (83.18% female), with an average age of 55.4 years (±13.9 years; minimum = 23 years; maximum = 82 years), who completed the entire PA program (Table 1). For most of them (83.19%; n = 94), the referral was made by their general practitioner. Diabetes and obesity represented more than half of beneficiaries, accounting for 27.43% and 24.78%, respectively. It is also important to note that 85.84% (n = 97) of the participants were overweight (BMI > 25 kg/m^2^) and two-thirds had a BMI > 30 kg/m^2^.

### 2.3. Procedure

Following the PAP, the patients were received by a professional in adapted PA in the local association. An initial assessment was carried out using the various measurement tools mentioned above, enabling an individualized PA program to be co-constructed with the patients. At the end of the 8-week program, a second assessment was carried out by the same professional in adapted PA. On this occasion, patients were referred to sports facilities suited to their abilities, to continue their physical and/or sports activities after leaving the BSO^®^ program. All participants included in this study (n = 113) completed at least 80% of the program (i.e., 12 out of 16 sessions) and were present at the initial and final evaluation sessions.

### 2.4. Measurements

#### 2.4.1. Anthropometric Data

To calculate BMI, participants’ height was measured using a measuring rod, and weight was measured using an analog scale. In line with the French National Authority for Health (HAS) recommendations [21], BMI measurement was complemented by waist circumference measurement using a tape measure.

#### 2.4.2. Physical Condition Measurements

To obtain an objective assessment of the participant’s physical condition and cardiorespiratory capacity, two walking tests were carried out. The 6 min walk test (6MWT) measures the walking perimeter of participants over 6 min. This scientifically validated field test is a sub-maximal effort test commonly used to measure functional capacity, particularly in the case of people with chronic illnesses. It has the advantage of being simple, safe, well tolerated, and representative of people’s daily activities [22]. When environmental conditions did not allow the 6MWT to be used (infrastructure too small, etc.), the 2 min walk-in-place test was preferred. This consists of a series of knee lifts, reaching a line halfway between the kneecap and the crest of the iliac bone (upper tip of the hip). Like the 6MWT, this scientifically validated field test [23] is particularly adapted to people with chronic diseases.

As a complement to the walking test, and to measure the participants’ muscular strength, the “30 s Chair Stand Test” was used. This test measures the number of maximum repetitions of chair lifts performed in 30 s. It is effective in measuring lower limb strength [23].

Finally, to measure the level of flexibility, a test consisting of standing with legs straight and bending the trunk by bringing the hands as low as possible (without bending the legs) was carried out. The distance between the fingers and the ground, or the degree to which the hands touch the ground, is used to define a form index (ranging from 1 to 5).

#### 2.4.3. Physical Activity

Firstly, for two years (2016–2017 and 2017–2018), PA levels were measured using the *Score d’Activité Physique de Dijon* [24,25]. This 9-item scale measure covers a general assessment of PA level (1 item), daily activities (2 items), sports and leisure activities (5 items), and rest time (1 item). The total score, with a maximum of 30 points, distinguishes very active people (score > 20) from non-active people (score < 10). During the last season (2018–2019), this questionnaire was replaced by the *Ricci et Gagnon questionnaire* used by the French social security system.

This questionnaire includes 9 questions assessing habits related to PA, Sedentary behavior (1 item), leisure activities (including sport) (4 items), and activities of daily life (4 items). Scores for each question range from 1 to 5 points and the total score ranges from 9 to 45 points.

The total score identifies three activity profiles: inactive (score < 18), active (score between 18 and 35), or very active (score > 35). This questionnaire was preferred to the previous one, which did not allow sedentary time to be quantified under the heading of “rest time”, as the latter included sleep time.

In this study, the level of physical activity was categorized as follows: inactive, active, and very active.

### 2.5. Program

The program proposed to participants aims to support sedentary, physically inactive people with a chronic condition by offering them an adapted PA (APA) program. It took place in groups (no more than 10 participants) over 8 weeks (evaluation sessions were scheduled before and after the program). It was supervised by a professional in APA. Several PAs were offered to participants (e.g., Nordic walking, aqua gym, gymnastics, stretching). Each participant benefited from two weekly one-hour sessions in one or more activities of their choice. The professional in APA paid particular attention to each participant’s level of intensity (moderate intensity was the goal). Alongside the supervised activities, participants were made aware of the health benefits of PA and the repercussions of a sedentary lifestyle through group health education workshops and were offered a range of motivational tools to increase their independent exercise. More specifically, they were invited to modify their lifestyle habits to make more room for PA in all acts of daily life (preferring stairs to elevators, using the car less, walking more, etc.) and to be less sedentary during the day. To achieve this, they were given a booklet in which they recorded their physical practice every day. During the final evaluation, this booklet was used to discuss any barriers and levers to changing habits, and to envisage new objectives for the continuation of autonomous or club sports.

### 2.6. Statistical Analysis

Firstly, descriptive analyses in terms of numbers, percentages, means, and standard deviations were carried out before the start of the program (T0) and at the end of the program, i.e., 8 weeks later (T1). Secondly, to decide which analyses to perform, we first checked the normality of the data for each variable using the Shapiro–Wilk test. Then, intra-group comparisons (paired Student’s *t*-test or Wilcoxon test and Chi^2^) were conducted to measure changes in our variables of interest between T0 and T1, according to the normality of the data.

Finally, with 85% of beneficiaries having a BMI greater than 25 kg/m^2^, the classification of patients for whom the program was most beneficial was based on anthropometric characteristics. Therefore, Pearson correlational analyses were performed between the deltas (difference between T1 and T0) of weight loss (delta weight), BMI (delta BMI), waist circumference (delta waist circumference), and different variables of interest at inclusion (e.g., age, waist circumference).

All analyses were performed using JASP Team (2023) software (Version 0.17.1).

## 3. Results

Concerning the first objective of this study, our results highlight a significant improvement in participants’ physical condition at the end of the program (H1), expressed in a significant increase in cardiorespiratory endurance (+14%; *p* = 0.001 with 2MWT and +8.55%; *p* < 0.001 with 6MWT), flexibility (+12.5%; *p* < 0.001), and muscular strength (+22.7%; *p* < 0.001; Table 2). To a lesser extent, our results also highlight an improvement in anthropometric data (hypothesis 2), illustrated by a significant reduction in weight (−0.71%; *p* < 0.001), BMI (−0.93%; *p* < 0.001), and waist circumference (−0.9%; *p* = 0.04).

**Figure 2:**
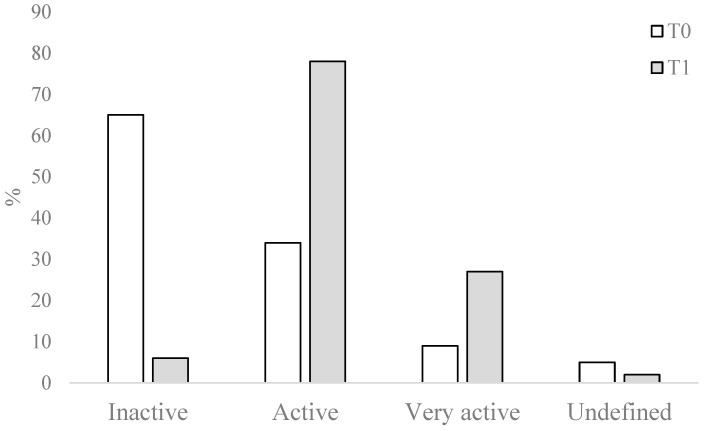
Physical activity behavior before and after the PAP (n = 113).

In addition, at the end of the program, we also noted a significant improvement in PA behavior (hypothesis 3) (Khi^2^ = 15.651; *p* = 0.004) with an increase in the level of PA (Figure 2), illustrated by a significant increase in the number of individuals in the “active” and “very active” profiles, and a significant reduction in the number of people in the “inactive” profile.

Given the considerable heterogeneity of our population, reflected in high standard deviations, we wished to identify patient characteristics at inclusion that could be associated with improved post-program anthropometric data, suggesting for whom the PA program had proved most beneficial. To measure program effectiveness and respond to objective 2 of this study, deltas of weight, BMI, and waist circumference (difference between T1 and T0) were used. Correlational analyses revealed that the decrease in BMI and weight throughout the program correlated positively with age (r = 0.252 and *p* = 0.007, and r = 0.247 and *p* = 0.008, respectively) and negatively with BMI from baseline (r = −0.271; *p* = 0.004) (Table 3). In other words, the older the participants and the lower their BMI at the start of the program, the greater the positive effects of the program on reducing their BMI and weight. Finally, delta waist circumference was negatively correlated with baseline waist circumference (r = −0.219; *p* = 0.022). In other words, the lower the starting waist circumference, the greater the loss at the end of the program.

## 4. Discussion

First of all, regarding the socio-demographic profiles of participants in France [13,26], as well as internationally [27,28,29,30], this type of health-related scheme attracts more women than men, which is confirmed in this study with 83.18% women.

The first objective of this study was to evaluate the effectiveness of the program set up in a community setting in France, with people benefiting from a PAP, in particular to compensate the lack of data on PA prescription in France. Firstly, in relation to hypothesis 1, our results show a significant improvement in participants’ physical condition, with up to 14% improvement in the distance covered in walking tests. This result is in line with those obtained by Reed and colleagues [31], who found, after a 12-week program, an improvement of nearly 14% in cardiorespiratory endurance for beneficiaries with coronary artery disease enrolled in Nordic walking and +10% for beneficiaries enrolled in the moderate-to-vigorous-intensity continuous training. Similar results can be explained by the aerobic activities included in the program, such as Nordic walking, which improves aerobic capacity and walking speed [32]. Furthermore, a meta-analysis comparing types of program shows that the programs based on aerobic exercise training shorter than 12 weeks (n = 244) improve cardiorespiratory fitness in adults with overweight and obesity [33].

In addition, our results highlight a significant improvement in flexibility (+12.5%) and muscle strength in the lower limbs (+29.7%). This is in line with an 8-week program with 50 elderly women, which showed a significant improvement in muscle strength in the lower limbs (+31.31%) with the 30 s Chair Stand test [34]. Another study on 19 hypertensive obese elders highlighted that 12 weeks of moderate-intensity aerobic training increased flexibility compared with baseline (+21.6%; *p* = 0.01) [35]. These improvements can be explained by the various activities included in the program, such as aqua gym, gymnastics, and stretching, which mainly improve muscle strength and flexibility [36,37,38].

In relation to hypothesis 2, our results show a significant reduction in participants’ weight (−0.71%), waist circumference (−0.88%), and BMI (−0.93%) at the end of the program. These three indicators are often used in studies of PA prescription [7] and are therefore important objectives in a PA program aimed at reducing the prevalence or incidence of non-communicable diseases, particularly as they are associated with an increased risk of cardiovascular disease, type 2 diabetes, and cancer [39,40,41]. It is important to note that the reduction in BMI was small (−0.3 kg/m^2^) in our study, but consistent with the mean reduction of 0.21 kg/m^2^ (equivalent to about 0.7% loss) reported in a meta-analysis of the same type of intervention [42]. These data are more encouraging given the short duration of our program (8 weeks).

In relation to hypothesis 3, our results also highlight an improvement in PA levels at the end of the program, illustrated by an increase in the number of active and very active people and a decrease in the number of inactive people, even though, at inclusion, nearly 58% of participants were inactive. Galaviz and collaborators [9] showed that women included in a PAP were more physically active after 8 weeks of community exercise since the proportion meeting Canadian PA guidelines increased from baseline to post-intervention from 17% to 50%. This result is consistent with a scoping review (n = 19 studies) on PAP involving a qualified PA professional which reported favorable impacts on patients’ subjectively measured PA after an exercise program and/or specific counseling [43]. If these PA behavior changes are maintained over time, it would help reduce participants’ risk of developing certain health problems such as coronary heart disease and diabetes [44]. In addition to the activities proposed, which naturally increased their level of PA, there is every reason to believe that all the interventions aimed at raising awareness of the harmful effects of a sedentary lifestyle, and the many motivational tips and tools presented to participants, could enable these levels to be maintained over time, even if this remains to be confirmed by longitudinal studies.

The second objective of this study was to identify the characteristics of patients at inclusion on whom the program would have the greatest beneficial effects. Correlational analyses show that the program was more effective for older participants, particularly in terms of BMI and weight. Correlational analyses also highlight that the program was more efficient for people who are overweight than for those who are obese. These results are in line with an UK review [45] which showed that programs linked to PA prescription increase PA levels in certain populations, namely elderly people, and those who are overweight (but not obese). A recent study on 647 participants showed that increasing age predicted an increased likelihood of completion, with greater participation in sessions and in the program as a whole [46]. As mentioned by Portman and collaborators [46], this may be due to the fact that older people have fewer time constraints, an increased desire for social interaction, and easier incorporation of PAP-related activities into daily life.

### 4.1. Bias and Limitations

This study has several limitations, which means that the results should be treated with a degree of caution. Firstly, women represent over 80% of our sample, which limits the generalizability of our results, particularly for men. This is partly explained by the fact that the prevalence of physical inactivity is higher in women than in men [47,48] and that women also participate in PA more often for reasons of health or physical appearance, whereas men participate more for pleasure and competition [49]. This corresponds to a study of 973 participants accessing the PAP scheme, 60.4% of whom were mainly women [46].

Our study shows another recruitment bias such as favoring urban to rural environments or lacking large segments of people [50]. In addition, some assessment tools (notably the walking test and the PA questionnaire) were modified depending on the years or contexts in which they were administered. While this implies that these results should be viewed with caution, these limitations are also imposed by problems of space limitations (for the walking test) and reservations about the reliability of self-reported tools for measuring PA [51]. To measure changes in PA and sedentariness, it would be interesting to couple objective measurements using accelerometry [52]. Lastly, the proposed study corresponded to a program carried out in the community to fulfill the expectations of the participants; the types of PA were not imposed, and the program was not restricted to one type of activity. As a result, participants were free to participate in any PA or sport of their choice. The total number of sessions attended was therefore tracked, but a differential analysis by activity type was not possible.

### 4.2. Perspectives

The final evaluation was conducted just after the end of the program, which does not allow us to conclude that the effects observed are maintained over time. It therefore seems important to continue research aimed at measuring the effects of PAP programs by diversifying the sample, by trying to balance the number of men and women, by carrying out measurements several months after the end of the program to apprehend the effects over time, and by coupling subjective measurements with objective measurements [53]. In addition, it is essential to extend investigations beyond the period of the COVID-19 pandemic, as our study focused on the 3 years following the implementation of the PAP in France, up to the pandemic. It is therefore essential to obtain post-COVID data in France, so that we may compare our findings with the international literature.

To conclude, the major challenge in the years of the future will be to ensure the translation of PA interventions between research and practice. It is essential to be able to evaluate the effectiveness of intervention programs while enabling them to be carried out in the community. Koorts and collaborators [5] mention that complex characteristics of real-world implementation settings and the multilevel barriers to effective implementation are two of the many challenges faced when translating evidence-based interventions into practice, it is therefore essential for all stakeholders to work in synergy for PA promotion.

### 4.3. Practical Applications

A team from the UK [54] presented the various phases of the PAP process in order to implement sustainable programs. It is necessary to (1) define the beneficiary public, the prescriber, and the type of PA prescribed; (2) define who will carry out the program and how it will be paid for; (3) take into account financing arrangements; (4) take into account individual and environmental constraints that may hinder the beneficiary’s participation in the program; and (5) measure the program’s effectiveness by considering multiple parameters (physical or clinical improvements, adherence rates, psychological parameters), in particular in order to anticipate the adoption of active behaviors beyond the program.

Currently, in France, only the first phase is partially defined. As mentioned in the methodology section, given the acute lack of data on PAP in France, this study was carried out retrospectively within a sports association applying a predefined evaluation protocol, mainly on characteristics related to physical conditions.

This study is therefore a first step in the deployment of PAP in France, with quantitative data to allow PAP professionals to improve or compare their practices.

## 5. Conclusions

In this study, we presented the first data on PA prescription in France, which only date from 2016, using data from a short 8-week community-setting PA program. The results suggest encouraging prospects for the future. Among the key findings, a short program among beneficiaries with a chronic condition supervised by qualified professionals (1) improves participants’ physical condition; (2) improves anthropometric parameters; and (3) modifies PA behavior. Furthermore, in the context of the program set up specifically for this purpose, it would appear that elderly and overweight people are more likely to exhibit beneficial effects on anthropometric parameters than younger participants or those with a high level of obesity.

The increase in chronic diseases has highlighted the need to develop more interventions aimed at promoting PA and reducing sedentariness, leading to the development of prescription PA. The scientific literature on schemes presents them as relevant tools, but PAP in France is a recent public policy, with very few funding resources allocated to prevention. It is therefore essential to analyze practices in order to implement tangible and effective measures. The literature on French schemes is almost non-existent, and this study not only provides encouraging results, but also makes it worthwhile to investigate on-the-ground data in this area. Our results confirm the short-term effectiveness of these programs, including increased self-reported PA, weight reduction, improved physical fitness through enhanced cardiovascular performance, and increased muscular strength and flexibility for people with chronic diseases, many of whom are sedentary and/or inactive. Our results also highlighted the more marked effects for older people and those with a lower BMI at program entry.

Further research is needed to better understand the effects of these programs over time and to identify the populations for whom they are most effective, whether by age, gender, or initial pathology. To achieve this, it is essential to use larger cohorts, which is challenging for longitudinal studies in the community.

## Figures and Tables

**Table 1:** Participant characteristics at inclusion.

Characteristics	Number (%) N = 113
Participants per year	
2016–2017	9 (7.97)
2017–2018	41 (36.28)
2018–2019	63 (55.75)
Gender	
Women	94 (83.18)
Men	19 (16.81)
Professionals who prescribed physical activity	
General practitioner	94 (83.19)
Specialist	10 (8.85)
Hospital physician	7 (6.20)
Other	2 (1.77)
Chronic diseases and conditions	
Diabetes	31 (27.43)
Obesity	28 (24.78)
Coronary heart diseases	15 (13.27)
Cancer	8 (7.08)
Hypertension	7 (6.19)
Arthritis	5 (4.42)
Depression	4 (3.54)
COPD	2 (1.77)
Scoliosis	2 (1.77)
Other *	7 (6.19)
Undefined	4 (3.54)
BMI (kg/m^2^)	
Normal weight (18.5–24.9)	16 (14.16)
Overweight (25–29.9)	22 (19.47)
Obesity class I (30–34.9)	43 (38.05)
Obesity class II (35–39.9)	20 (17.70)
Obesity class III (≥40)	12 (10.62)
Physical activity level	
Inactive	65 (57.52)
Active	33 (29.20)
Very active	10 (8.85)
Undefined	5 (4.42)

COPD: Chronic Obstructive Pulmonary Disease; * Other: n = 1 per pathology (back pathology, hypercholesterolemia, multiple sclerosis, polyneuropathy, peripheral artery disease, pre-diabetes, asthma).

**Table 2:** Mean comparisons before (T0) and after (T1) the program (N = 113).

Variables	T0	T1	*p*
Weight (kg)	84.9 (17.1)	84.3 (16.8)	<0.001
BMI (kg/m^2^)	32.3 (5.9)	32.0 (5.8)	<0.001
Waist circumference (cm)	102.6 (13.7)	101.7 (13.4)	0.04
6MWT (m) ^1^	474.9 (64.5)	515.5 (57.6)	<0.001
2MWT (m) ^2^	78.1 (19.4)	89 (19.3)	<0.001
Strength	11.9 (3.6)	14.6 (4.9)	<0.001
Flexibility test	2.4 (1.2)	2.7 (1.2)	<0.001

Note: ^1^ n = 66; ^2^ n= 47 depending on the space available to carry out the test on the site.

**Table 3:** Pearson correlation matrix between variation in BMI, weight, waist circumference, and the other variables studied.

Variable	Delta* BMI		Delta* Weight		Delta* Waist Circumference	
	*r*	*p*	*r*	*p*	*r*	*p*
1. T0 Age	0.25	0.007	0.25	0.008	0.16	0.089
2. T0 Height	0.16	0.086	0.13	0.166	0.13	0.190
3. T0 Weight	−0.18	0.058	−0.20	0.036	−0.09	0.339
4. T0 BMI ^1^	−0.27	0.004	−0.27	0.004	−0.16	0.098
5. T0 Waist C. ^2^	−0.14	0.146	−0.15	0.108	−0.22	0.022
6. T0 6MWT ^3^	0.10	0.420	0.11	0.368	0.19	0.139
7. T0 2MWT ^4^	0.11	0.494	0.09	0.574	0.24	0.112
8. T0 Strength ^5^	−0.14	0.173	−0.12	0.249	−0.07	0.492

* Deltas correspond to the difference between T1 and T0 results. ^1^ Body Mass Index; ^2^ waist circumference; ^3^ 6 min walk test; ^4^ 2 min walk test; ^5^ 30 s Chair Stand Test.

## Data Availability

The data presented in this study are available on request from the corresponding author.
